# Birthweight trends and their explanatory factors in Hungary between 1999 and 2018: an analysis of the Hungarian Tauffer registry

**DOI:** 10.1186/s12978-024-01787-0

**Published:** 2024-04-12

**Authors:** László Zsirai, Attila Kun, Gergely Á. Visolyi, Márk M. Svébis, Beatrix A. Domján, Ádám Tabák

**Affiliations:** 1 Department of Gynaecology and Family Planning, Istenhegyi GeneDiagnostic Center, Budapest, Hungary; 2 Department of Obstetrics and Gynaecology, Tolna County Balassa János Hospital, Szekszárd, Hungary; 3 Outpatient Department of Obstetrics and Gynecology, Paks Health Centre, Paks,, Hungary; 4 Károly Rácz School of PhD Studies, Semmelweis University, Budapest, Hungary; 5 Department of Internal Medicine and Oncology, Semmelweis University Faculty of Medicine, 26 Üllői Str., Budapest, H-1085 Hungary; 6 Bajcsy-Zsilinszky Teaching Hospital, Budapest, Hungary; 7 UCL Brain Sciences, University College London, London, UK; 8 Department of Public Health, Semmelweis University Faculty of Medicine, Budapest, Hungary

**Keywords:** Birthweight, Caesarean section, Gestational age, Labor induction, Maternal age, Obstetrical database, Parity, Population-based study, Pregnancy, Week of delivery

## Abstract

**Background:**

The increasing birthweight trend stopped and even reversed in several high income countries in the last 20 years, however the reason for these changes is not well characterized. We aimed to describe birthweight trends of term deliveries in Hungary between 1999 and 2018 and to investigate potential maternal and foetal variables that could drive these changes.

**Methods:**

We analysed data from the Hungarian Tauffer registry, a compulsory anonymized data collection of each delivery. We included all singleton term deliveries in 1999–2018 (*n* = 1,591,932). We modelled birthweight trends separately in 1999–2008 and 2008–2018 in hierarchical multiple linear regression models adjusted for calendar year, newborn sex, maternal age, gestational age at delivery, and other important determinants.

**Results:**

Median birthweights increased from 3250/3400 g (girl/boy) to 3300/3440 g from 1999 to 2008 and decreased to 3260/3400 g in 2018. When we adjusted for gestational age at delivery the increase in the first period became more pronounced (5.4 g/year). During the second period, similar adjustment substantially decreased the rate of decline from 2.5 to 1.4 g/year. Further adjustment for maternal age halved the rate of increase to 2.4 g/year in the first period. During the second period, adjustment for maternal age had little effect on the estimate.

**Conclusions:**

Our findings of an increasing birthweight trend (mostly related to the aging of the mothers) in 1999–2008 may forecast an increased risk of cardiometabolic diseases in offsprings born in this period. In contrast, the decreasing birthweight trends after 2008 may reflect some beneficial effects on perinatal morbidity. However, the long-term effect cannot be predicted, as the trend is mostly explained by the shorter pregnancies.

## Abstract (plain language summary)

Birthweights showed an increase followed by a decrease in several high income countries in the last 20 years, however the reasons for these changes is not well described. Thus, we aimed to investigate birthweight trends and their potential explanatory factors in Hungary between 1999 and 2018. We used registry data of all deliveries from Hungary in 1999–2018 (*n* = 1 591 932). Birthweights increased from 3250/3400 g (girl/boy) to 3300/3440 g from 1999 to 2008 and decreased to 3260/3400 g until 2018. Maternal age explained approximately half of increase in the first period, while a substantial part of the decrease in the second period was explained by the presence of shorter pregnancies. The increasing birthweights in 1999–2008 may forecast an increased risk of cardiometabolic diseases in offsprings born in this period. In contrast, the decreasing birthweight trends after 2008 may reflect some beneficial effects on perinatal morbidity. However, its long-term consequences cannot be predicted, as the trend is mostly explained by the shorter pregnancies.

## Background

Available evidence suggests that both low and high birthweights of term infants are major negative determinants of newborn survival [1], while large infants are also more prone to injuries related to traumatic deliveries [2]. Similarly, there is some evidence for the association between both small and large for gestational age and the risk of an adverse cardiometabolic risk profile in childhood and common chronic diseases (such as cardiometabolic, neurological, immunological, gastrointestinal, and malignant disorders) in adulthood [3–5].

Given the strong association between birthweight and later chronic diseases, even small temporal changes in the distribution of term newborns’ birthweights could be of utmost public health importance. Indeed, an upward birthweight trend was observed in several high income countries and regions (such as the United States (US) [6], Canada [6, 7], the United Kingdom (UK) [8, 9], Norway [10], Sweden [11], Denmark [12], France [13], Australia [14], Croatia [15], Poland [16], and the Faroese Island [17]) at the end of the last century. In contrast, a reverse trend was found in Japan [18, 19] and the increase appeared to reverse in the US [20–25], China [26, 27], Portugal [28], Norway [29, 30], and Germany [31] after the 1990s. In a previous analysis of the Hungarian Tauffer database we observed a similarly increasing birthweight trend of term infants between 1996 and 2000, followed by a slight decrease until 2015, however we did not look for potential explanations of this phenomenon [32]. To the best of our knowledge, there is no whole population-based analysis on birthweight trends from Hungary although results from a tertiary care centre in Szeged show an increasing birthweight trend between 1989 and 2009 [33].

 While birthweight changes are well described in the literature, potential explanatory factors are much less known and these factors explain only parts of the slope of the birthweight trajectories. Most studies suggest that the increasing trends are associated with older maternal age [8, 9, 12–14, 19, 33], increasing maternal body mass index (BMI) [11–14] and height [15, 19], longer gestations [10, 12, 13, 17, 19], decreases in smoking [11–14], decreasing parity [13, 15, 17], changes in ethnicity [9] and socioeconomic factors [9, 15], while the decreases could be related to decreases in the length of gestation [18–20, 22, 25, 28], induction of labour [6, 20, 22, 24, 25], and early term caesarean sections [6, 20, 22, 24], increases in primiparity [18, 19], and decreased foetal growth [22, 23].

The purpose of the present analysis was to (1) extend our previous birthweight trend analysis until 2018 and (2) to investigate potential maternal and foetal variables (including common pathologies) that could drive these changes using data from the Hungarian Tauffer registry of all pregnancies.

## Methods

### Setting and study design

The current study is a cross-sectional registry study of all term deliveries in Hungary between 1999 and 2018. We utilize the Tauffer database, which includes data from the compulsory report of each delivery in Hungary. After each parturition (24–43 weeks of gestation), an anonymized standardized report form is filled in and then collected by the National Healthcare Service Centre [32]. 

A detailed description of the database was published previously [32, 34–36]. In short, the nationwide Hungarian obstetrics database (“Obstetrics Regulation”) was initiated by Vilmos Tauffer in the early 1930s. The variables collected were standardized and extended in 1993. The Tauffer database was managed by the National Institute of Obstetrics and Gynaecology until 2010 when it was succeeded by the National Institute for Quality and Organizational Development in Healthcare and Medicines (ref. 76/2004 ESzCsM, Decree on the Determination, Collection, Analysis of Health-related Unidentifiable data; Ministry of Health Social and Family Affairs, Hungary). To comply with privacy regulations, the database contains anonymized records, which means that repeated deliveries by the same woman cannot be identified.

For the period covered in the current analysis (01/January/1999–31/December/2018), the Tauffer database contains 1,784,654 live births (94.8%) of the 1,881,437 live births recorded by the Hungarian Central Statistical Office [37].

The current analysis uses only unidentifiable information collected according to Hungarian law in agreement with European ethical directives. Thus, no ethical approval or individual consent was required for this analysis.

### Participants

**Fig. 1:**
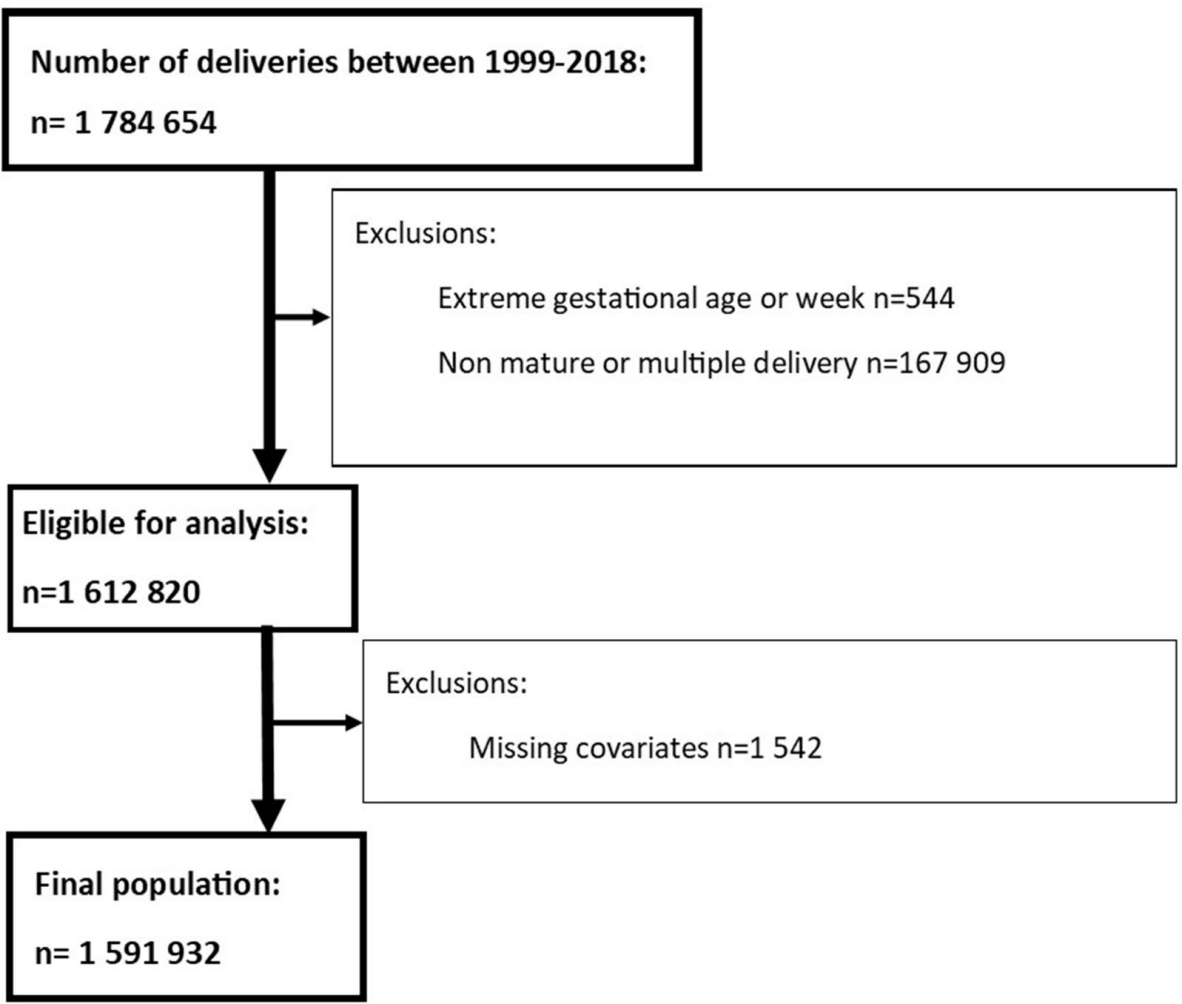
Flow-chart for the selection of study participants

Of the 1,784,654 deliveries we excluded non-term deliveries (< 37 or > 41 weeks of gestation), stillbirths, and multiple deliveries leaving 1,612,820 records eligible for analysis. We further excluded records with missing birthweights and covariates as well as those with extreme (likely erroneously recorded) birthweights leading to a final analytical sample of 1,591,932 (98.7% of those eligible) deliveries [14] (Fig. 1).

### Outcomes

The main outcome of the current analysis is birthweight (a mandatory field in the database) measured in grams (g) immediately after delivery on a calibrated scale according to WHO recommendation.

### Covariates

*Date of delivery* – we used year of each delivery as the major covariate of interest in our analyses.

*Maternal age* was calculated as the difference between the date of delivery and the date of the mother’s birth in years. Furthermore, we created a categorical variable of age for the interaction analysis to investigate whether changes in birthweight differentially affected mothers of younger, usual or advanced ages (< 25 years, 25–34 years, and ≥ 35 years).

*Gestational age at delivery* (a mandatory field in the database) was based on the woman’s last normal menstrual period if it coincided within 1 week of the date determined by crown-rump length determined by ultrasound done between 10 and 13 weeks of gestation, otherwise we used the ultrasound estimates [38, 39].

*Newborn sex* (a mandatory field in the database) is extracted from the discharge report and is based on the phenotype at birth.

*Maternal medical history* was recorded by the treating physician at delivery. For the present analysis, we included parity (number of living children).

*Obstetrical interventions* include data on the initiation of labour (spontaneous / induced) as well as the mode of delivery (coded as vaginal or caesarean section).

### Statistical analysis

First, we visually investigated the time trends of birthweights by newborn sex using loess curves. We found an increasing trend from 1999 with peak birthweights in 2008 followed by a decreasing trend until the end of the observation period. To improve the interpretation of models describing birthweight trends, we modelled the period with increasing (1999–2008) and decreasing trends (2008–2018) separately.

For descriptive purposes, we selected deliveries in 1999 (lowest birthweight from the first period), 2008 (peak birthweight), and 2018 (lowest birthweight in the second period). For the comparison of different variables in the selected years, chi2-tests for categorical variables and one-way analysis of variance (ANOVA) for continuous variables were used.

Then we modelled birthweight with multiple linear regression using calendar year and newborn sex as predictors (*Model 0*). In subsequent models we serially adjusted for other important predictors of birthweight. *Model 1* was further adjusted for gestational age at delivery, *Model 2* for maternal age, and *Model 3* for other important determinants (parity, delivery induction, and mode of delivery). For these models date of delivery was centred at 2008, maternal age at 29 years, and gestational age at 39 weeks. In separate linear regression models, we investigated whether the inclusion of quadratic or cubic terms of gestational age at delivery and maternal age would improve the prediction of birthweight. Based on these models, we used the linear and quadratic terms to adjust for the effect of maternal age, and the linear, quadratic, and cubic terms for the effect of age at delivery.

Finally, we looked for interactions between calendar year and selected parameters in separate models by adding a calendar year by the given variable interaction to *Model 3*. For this analysis, maternal age was categorized (< 25 years, 25–34 years, and ≥ 35 years). We decided to use this parameterization, so the interactions would be easier to interpret for the non-specialist readers. Finally, we calculated estimated marginal means from the interaction models for all those variables where a potential interaction was likely (*p*-value for interaction < 0.10) and showed them graphically with their respective 95% confidence intervals (CI).

All analyses were done using Statistical Package for the Social Sciences (SPSS 25.0) software. Two-tailed P values of < 0.05 were considered statistically significant.

## Results

### Loess curves of birthweight over time

**Fig. 2:**
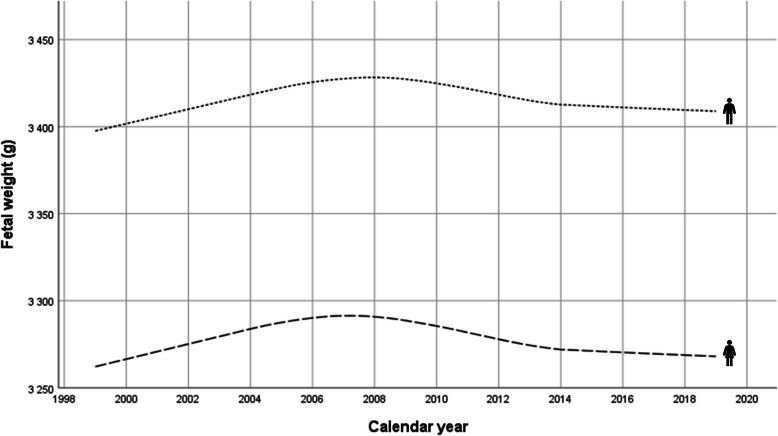
Temporal changes of mean birth weight by newborn sex in Hungary between 1999 and 2018. Loess curves

Mean birthweight increased almost linearly in both sexes by approximately 30 g in 1999–2008, followed by a faster decrease in 2008–2013 and a shallower decrease thereafter reaching a value within 10 g of the baseline in 1999 (Fig. 2).

### Foetal, maternal, and delivery related characteristics of pregnancies in 1999, 2008, and 2018

While there was no change in the sex distribution of newborns with around 51–52% of boys, all other parameters showed significant increasing or decreasing trends over the three selected years. Median birthweights were 3250/3400 g (girl/boy) in 1999, then increased to 3300/3440 g in 2008 and decreased to 3260/3400 g in 2018 (Table 1).

**Table 1:** Characteristics of singleton live births in Hungary in three selected years (1999, 2008, and 2018)

		1999	2008	2018	*P*-value
**Foetal parameters**					
Sex	n (%)				NS
	boys	38,167 (51.8)	43,855 (51.8)	40,488 (51.4)	
	girls	35,523 (48.2)	40,732 (48.2)	38,282 (48.6)	
Median birth weight	g				
	boys	3400 (3100;3700)	3440 (3130;3750)	3400 (3100;3700)	< 0.0001
	girls	3250 (2980;3550)	3300 (3000;3600)	3260 (3000;3550)	< 0.0001
**Maternal parameters**					
Age	year	26.2 (23.0;29.8)	29.6 (25.9;32.9)	30.5 (26.1;34.6)	< 0.0001
Age	n (%)				< 0.0001
	< 20 years	6087 (8.3)	5263 (6.2)	4625 (5.9)	
	20-24.9 years	23,940 (32.6)	12,691 (15.0)	11,378 (14.5)	
	25-29.9 years	25,854 (35.2)	26,991 (32.0)	20,708 (26.3)	
	30-34.9 years	12,819 (17.4)	28,718 (34.0)	23,752 (30.2)	
	35-39.9 years	3916 (5.3)	9179 (10.9)	14,038 (17.9)	
	≥ 40 years	915 (1.2)	1574 (1.9)	4117 (5.2)	
Parity	n (%)				< 0.0001
	primiparous	34,215 (46.4)	40,556 (47.9)	39,077 (49.6)	
	multiparous	39,475 (53.6)	44,031 (52.1)	39,693 (50.4)	
**Delivery-related parameters**					
Time of delivery	week	39.4 (38.5;40.1)	39.2 (38.4;40.0)	39.1 (38.3;39.9)	< 0.0001
Mode of delivery	n (%)				< 0.0001
	vaginal	60,740 (82.4)	60,320 (71.3)	47,224 (60.3)	
	caesarean section	12,950 (17.6)	24,267 (28.7)	31,108 (39.7)	
Induced delivery	n (%)				< 0.0001
	no	64,348 (87.3)	71,002 (83.9)	58,103 (73.8)	
	yes	9342 (12.7)	13,585 (16.1)	20,667 (26.2)	

Results are given as n (%) or median (IQR)

*IQR* Interquartile range

*P*-values are given for χ^2^-tests for categorical variables, and one-way ANOVA for continuous variables

Maternal age increased from 26.2 years in 1999 to 29.6 in 2008 and further to 30.5 in 2018. The proportion of older mothers (≥ 30 years of age) continuously increased from 24 to 53%. The proportion of primiparas increased from 46.4 to 49.6% while the frequency of multiparity decreased (Table 1).

Mean gestational age at delivery decreased by > 1 day between 1999 and 2018. The proportion of both induced deliveries and Caesarean sections more than doubled from 12.7 to 26.2% and 17.6 to 39.7%, respectively (Table 1).

### The role of foetal, maternal, and delivery related variables in the temporal changes of newborn birthweights

According to *Model 0*, birthweight significantly increased by 4.1 g/year in boys and girls in 1999–2008, while decreased by 2.5 g/year in 2008–2018 (Table 2).

**Table 2:** Hierarchical linear regression predicting birthweight (grams) of term newborns for the period 1999–2008 and 2008–2018

	1999–2008				2008–2018			
	Beta	SE	95% CI	*P*-value	Beta	SE	95% CI	*P*-value
**Model 0**								
Intercept	3300	0.98	3298–3302	< 0.0001	3291	1.06	3289–3293	< 0.0001
Calendar year (year)	4.14	0.17	3.80–4.47	< 0.0001	-2.48	0.15	-2.77-(-2.19)	< 0.0001
Boy	136.1	0.99	134.1–138.0	< 0.0001	140.4	0.94	138.6-142.3	< 0.0001
**Model 1**								
Intercept	3297	1.00	3295–3299	< 0.0001	3288	1.06	3286–3290	< 0.0001
Calendar year (year)	5.41	0.16	5.10–5.73	< 0.0001	-1.42	0.14	-1.68-(-1.15)	< 0.0001
Boy	141.5	0.92	139.7-143.3	< 0.0001	144.8	0.87	143.1-146.5	< 0.0001
Week of delivery (week)	145.0	0.89	143.3-146.8	< 0.0001	144.8	0.85	143.2-146.5	< 0.0001
(week of delivery)^2^	-19.85	0.34	-20.51-(-19.18)	< 0.0001	-18.46	0.33	-19.10-(-17.82)	< 0.0001
(week of delivery)^3^	1.78	0.28	1.22–2.33	< 0.0001	2.50	0.27	1.97–3.04	< 0.0001
**Model 2**								
Intercept	3319	1.04	3317–3321	< 0.0001	3304	1.08	3301–3306	< 0.0001
Calendar year (year)	2.36	0.16	2.05–2.68	< 0.0001	-1.81	0.14	-2.08-(-1.55)	< 0.0001
Boy	141.2	0.91	139.4–143.0	< 0.0001	144.9	0.86	143.2-146.6	< 0.0001
Week of delivery (week)	143.3	0.42	141.6-145.1	< 0.0001	145.3	0.83	143.7–147.0	< 0.0001
(week of delivery)^2^	-18.91	0.33	-19.57-(-18.25)	< 0.0001	-17.14	0.32	-17.77-(-16.51)	< 0.0001
(week of delivery)^3^	1.80	0.28	1.25–2.34	< 0.0001	1.95	0.27	1.42–2.48	< 0.0001
Maternal age (year)	9.41	0.09	9.30–9.65	< 0.0001	11.64	0.07	11.50-11.79	< 0.0001
(maternal age)^2^	-0.91	0.01	-0.94-(-0.89)	< 0.0001	-0.71	0.01	-0.73-(-0.69)	< 0.0001
**Model 3**								
Intercept	3296	1.21	3294–3298	< 0.0001	3274	1.22	3271–3276	< 0.0001
Calendar year (year)	2.62	0.16	2.30–2.93	< 0.0001	-1.82	0.14	-2.09-(-1.56)	< 0.0001
Boy	141.0	0.91	139.3-142.8	< 0.0001	144.8	0.86	143.1-146.5	< 0.0001
Week of delivery (week)	144.2	0.88	142.5–146.0	< 0.0001	148.0	0.84	146.4-149.6	< 0.0001
(week of delivery)^2^	-18.62	0.34	-19.28-(-17.97)	< 0.0001	-16.49	0.32	-16.13-(-15.86)	< 0.0001
(week of delivery)^3^	1.81	0.28	1.26–2.36	< 0.0001	1.68	0.27	1.15–2.21	< 0.0001
Maternal age (year)	8.30	0.09	8.12–8.48	< 0.0001	10.43	0.08	10.28–10.59	< 0.0001
(maternal age)^2^	-0.90	0.01	-0.92-(-0.88)	< 0.0001	-0.71	0.01	-0.73-(-0.69)	< 0.0001
Multiparous	35.97	0.96	34.09–37.85	< 0.0001	47.81	0.90	46.04–49.57	< 0.0001
Induced delivery	0.25	1.49	-2.67-3.17	NS	10.52	1.27	8.04–10.01	< 0.0001
Caesarean section.	13.99	1.25	11.54–16.43	< 0.0001	11.57	1.08	9.45–13.69	< 0.0001

For these models date of delivery was centred at 2008, maternal age at 29 years, and gestational age at 39 weeks

(week of delivery)^2^ and (week of delivery)^3^ refer to the quadratic and cubic terms of week of delivery. (maternal age)^2^ refers to the quadratic term of maternal age

*SE* Standard error, *CI *Confidence interval

When we adjusted for gestational age at delivery (including linear, quadratic and cubic terms; *Model 1*) the rate of increase in the first period became even more pronounced (5.4 g/year). During the second period, similar adjustment for gestational age at delivery substantially decreased the rate of decline from 2.5 to 1.4 g/year (Table 2).

Further adjustment for maternal age (including linear and quadratic terms; *Model 2*) halved the rate of increase in birthweight from 5.4 to 2.4 g/year. During the second period, adjustment for maternal age somewhat increased the estimate of yearly change in birthweight (Table 2).

Our final model (further adjusted for parity, induced deliveries, and caesarean sections; *Model 3*) showed similar estimates to the ones in *Model 2* (Table 2).

### Interaction between selected maternal, foetal, and delivery related characteristics and calendar year

**Fig. 3:**
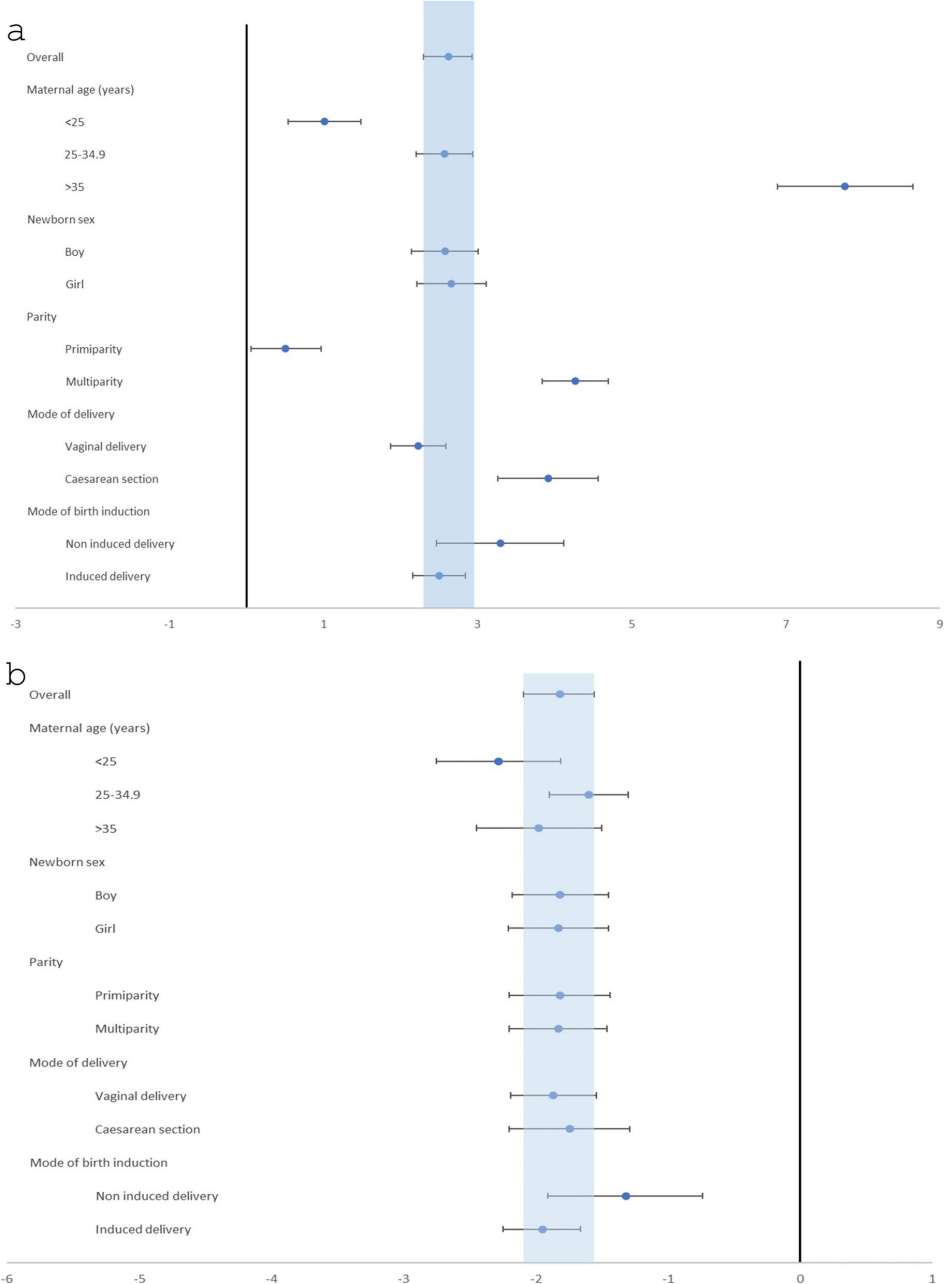
Yearly changes in birthweight of term newborns in 1999–2008 (**a**) and 2008–2018 (**b**). Birthweights in grams. All models are adjusted for gestational age at delivery (using linear, quadratic and cubic terms), maternal age (using linear and quadratic terms), parity, induced delivery, and caesarean section. Multiple linear regression. See further details in the Statistical Analysis section

**Fig. 4:**
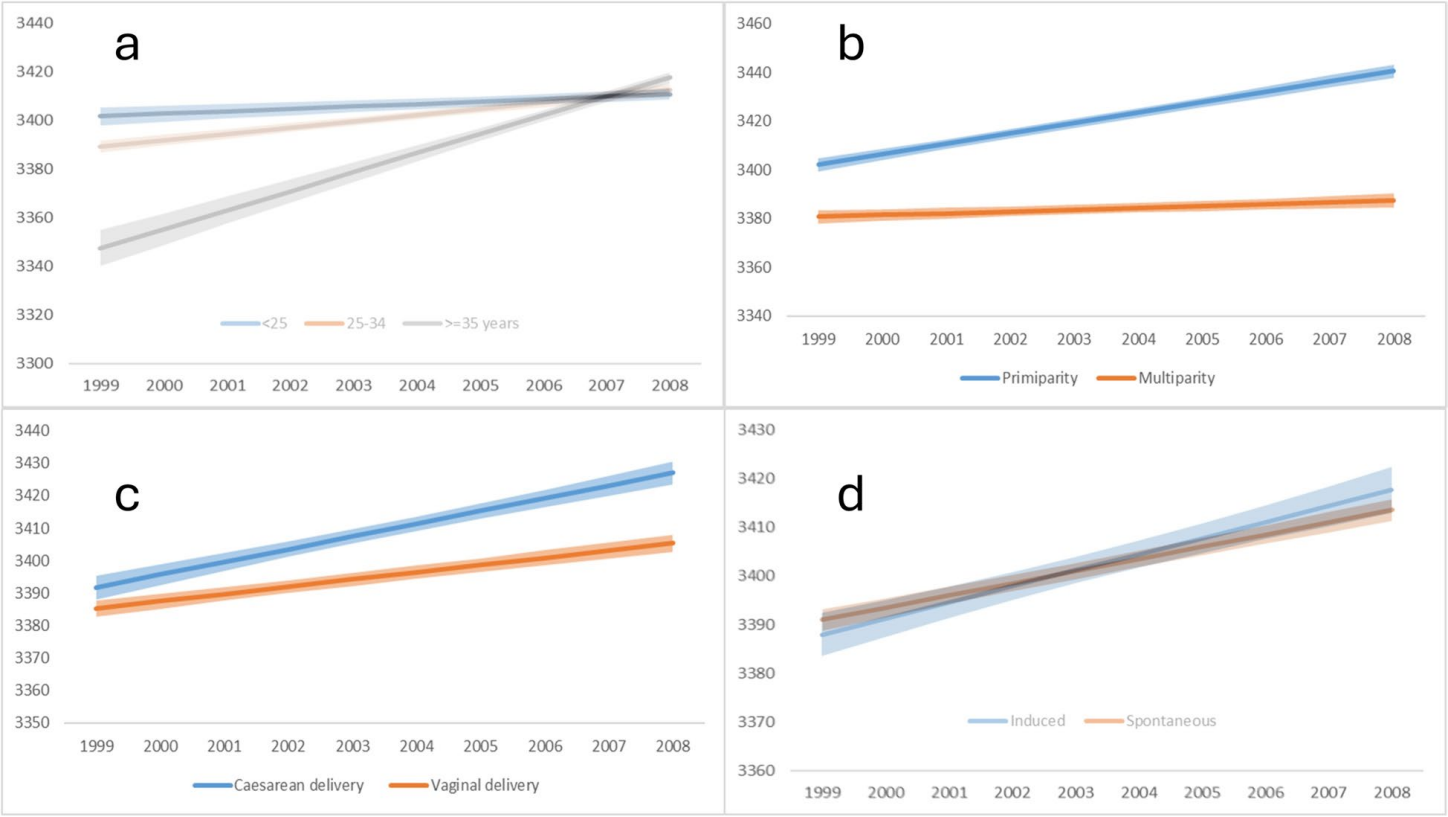
Birthweights by maternal age (**a**), parity (**b**), mode of delivery (**c**), and mode of birth induction (**d**) in 1999–2008. Birthweights in grams, shaded areas represent 95% confidence bands. Estimated marginal means for singleton term deliveries with the following characteristics: 48% female newborns, 47% primiparas, 76% vaginal deliveries, 85% non-induced deliveries, maternal age 28.0 years, gestational age at delivery 39.2 weeks. See further details on the modelling approach in the Statistical Analysis section

In the first period (1999–2008), we found a significant interaction between calendar time and maternal age (*p* < 0.0001), showing the fastest increase in birthweight of mothers over 35 years of age (vs. a slower increase in both groups of younger mothers) leading to similar birthweights in all age groups by 2008. Similarly, there was a strong interaction with parity, with widening birthweight gap between multiparous and nulliparous women (*p* < 0.0001) resulting from a slower increase in nulliparous and a faster increase in multiparous women. The mode of delivery was also related to the temporal increase in birthweights with a faster increase among those born by caesarean section (*p* < 0.0001). No interaction between newborn sex (*p* = 0.801) or the mode of induction (*p* = 0.080) with calendar time on birthweights was found (Figs. 3A and 4).

**Fig. 5:**
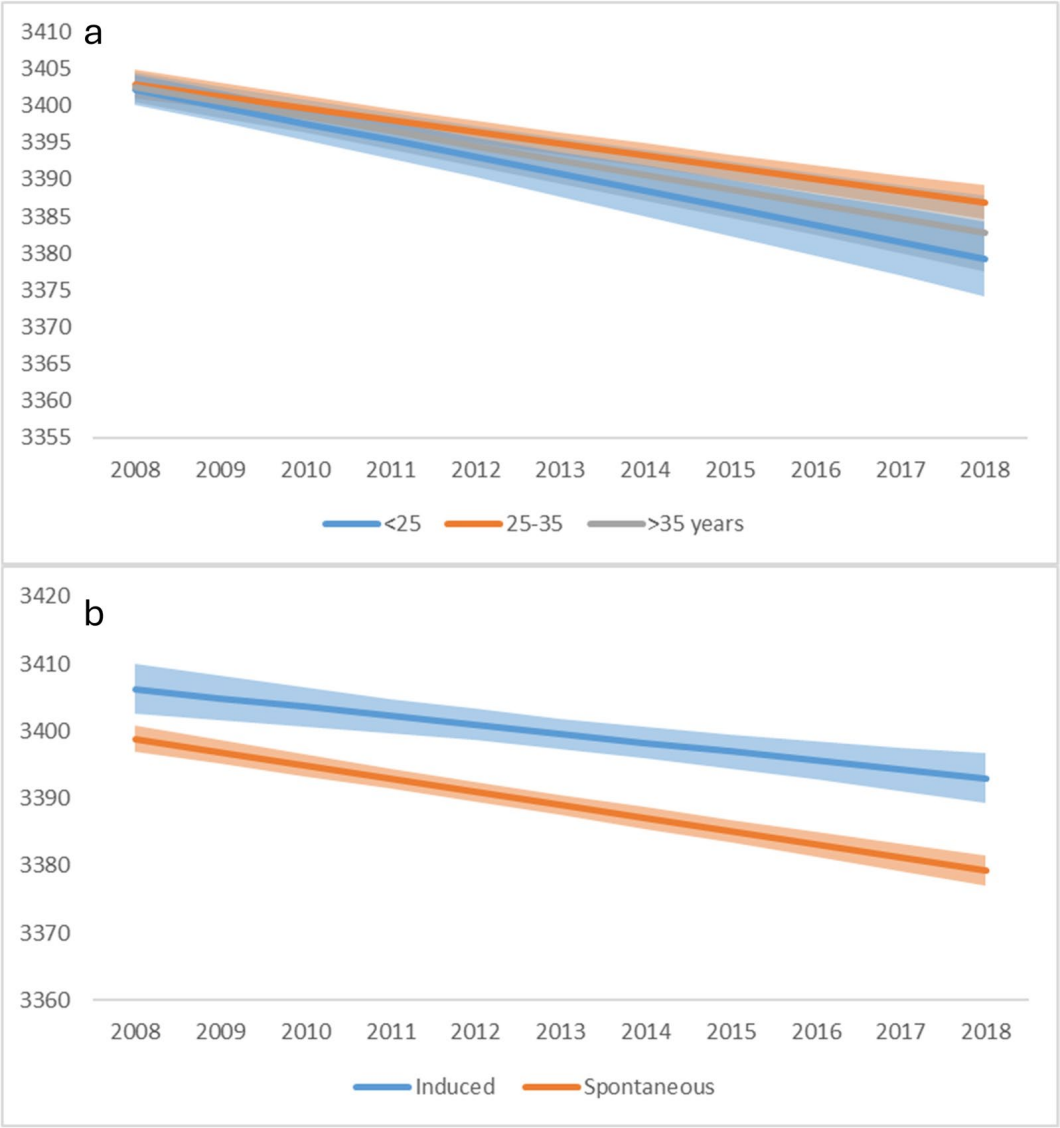
Birthweights by maternal age (**a**), and mode of birth induction (**b**) in 2008–2018. Birthweights in grams, shaded areas represent 95% confidence bands. Estimated marginal means for singleton term deliveries with the following characteristics: 48% female newborns, 49% primiparas, 66% vaginal deliveries, 80% non-induced deliveries, maternal age 29.9 years, gestational age at delivery 39.1 weeks. See further details on the modelling approach in the Statistical Analysis section

In the second period (2008–2018), we found a significant interaction with maternal age (*p* < 0.009), however the direction of the interaction was the opposite compared to the previous period: newborns of the youngest mothers showed the fastest decline in birthweight over time. The interaction with parity (*p* < 0.773) also changed, both primiparas and multiparas had a similar decrease in birthweights over time. Similarly to the first period, no interaction with sex of the newborn (*p* < 0.948) was found. Furthermore, the rate of decrease in birthweight was similar in both types of deliveries (*p* < 0.672) and was independent of presence or absence of induction (*p* < 0.059) (Figs. 3B and 5).

## Discussion

### Interpretation of main findings

An analysis of almost all full-term births in Hungary in 1998–2018, clearly showed an increasing birthweight trend of 4.1 g/year until 2008, followed by a less steep decline of 2.5 g/year in 2008–2018. During the same period, important changes in maternal and delivery related characteristics were observed: gestational age at delivery decreased, maternal age increased, the proportion of first parities, the frequency of both caesarean sections and induced deliveries increased.

According to our multivariate models, most of the increase in birthweight in the first period was explained by the increasing maternal age, while a substantial part of the decrease in the second period was explained by decreasing duration of pregnancies (i.e., decreasing gestational age at delivery).

When we investigated interactions between pregnancy related factors and calendar time (i.e., subgroups with the least and most changes over time), we found that the most pronounced difference between the first and second period was in mothers over 35 years of age, who had the fastest increase in the first period followed by a decrease similar to that of the younger age groups and the mean yearly change. Furthermore, the increase of birthweights in the first period was faster in newborns delivered by caesarean sections compared to vaginal deliveries, however no such interaction in the second period was found. Similarly, the increase in birthweights in the first period was more pronounced in multiparas compared to primiparas, while no interaction by parity in the second period was found.

### Validity of results

#### Birthweight trends

The increasing birthweight trend observed in the first period (1999–2008) parallels with similar observations from other high income countries [6–14, 17] including those from Croatia [15], Poland [40] and a regional database analysis from Hungary [33].

During the second period we found declining birthweight trends. This is in line with observations from the U.S., where the average birthweight of term pregnancies declined from 3,315 g in 1990 to 3,247 g in 2013, a decrease of 67 g [24]. The validity of this observation was confirmed by other reports from Japan [18, 19], the U.S [20–25]. , Norway [29, 30], Portugal [28], China [26, 27], Chile [41], and Germany [31]. Overall, a similar decrease to the one observed in Hungary was also found in most developed countries, however the decrease started mostly a decade earlier than in Hungary. In contrast, birthweights did not change significantly in low and middle-income countries from Africa, Asia and Central America between 2013 and 2018 [42].

#### Decreasing gestational age at delivery

Gestational age at delivery declined by two days between 1999 and 2018. This trend is similar to other surveys, however the magnitude of the decline varies between less than 1 to almost 3 days between 1990 and 2013 in the different studies [20, 24, 25, 31, 43–45]. Furthermore, there is evidence at least from the US that the decreasing gestational age at delivery is driven by labour inductions and early term caesarean deliveries [43].

#### Increasing maternal age over time

We found that median maternal age at delivery increased from 26.2 years in 1999 to 30.5 years in 2018, corresponding to an increase in the proportion of older mothers (≥ 30 years) from 24 to 53%. An increasing trend in maternal age is reported from most countries worldwide [14, 46]. For example, the mean age of primiparas increased from 24.9 years to 26.3 years in the U.S. between 2000 and 2014 [47].

#### Decreasing parity over time

During the 20-year observation period, the proportion of primiparas increased from 46.4 to 49.6%. Our results are somewhat different from those in other developed countries. For example, the proportion of primiparity remained constant (43.3%) in France between 1998 and 2003 [13], while it decreased (37.3–33.7%) in the US between 2000 and 2008 [23].

#### Increasing rates of caesarean sections and induced deliveries

The rate of caesarean sections and labour inductions more than doubled (from 17.6 to 39.7% and from 12.7 to 26.2%, respectively) in Hungary between 1999 and 2018. This is in line with observations from almost all countries. The rate of scheduled or induced deliveries almost tripled reaching over 30% in the US between 1990 and 2013 [23, 24, 43]. Similar, but smaller increase (25.9–33.6%) was observed in Scotland in 1988–2012 [48]. The rate of caesarean sections increased in the US [43] and similarly in India (from 28.2 to 42.0% in 2010–2017) [49] and Brazil (from 34.1 to 57% in 1997–2014) [50, 51], while the increase was minimal in Norway (13.6–16.3% in 1999–2016) [52].

#### Potential explanation for the increasing birthweight trends in the first period

According to our hierarchical logistic regression models, maternal age explained a large proportion (5.4 g/year vs. 2.4 g/year – 55.5%) of the increasing birthweight trend over time. This is in agreement with findings from other studies from high-income countries [8, 9, 12–14, 19, 33].

While maternal age may be directly related to birthweight, it could be a marker of other determinants, such as anthropometric, lifestyle or social factors that are also reported to be related to the increasing birthweight trends [9, 11–15, 19, 41]. For example, maternal smoking might decrease with maternal age [9, 12]. Similarly, maternal weight increases with aging and maternal BMI is a known predictor of newborn weight [53]. Indeed, there is an increasing trend in obesity among fertile aged women in Hungary in the last decades [54]. Furthermore, older age is associated with better socioeconomic circumstances that is associated with larger birthweights [55]. As advanced maternal age is also associated with higher risk of adverse obstetrical and perinatal outcomes [56], as well as elective deliveries [23] the changes observed during the first period could be associated with worsening pregnancy outcomes.

#### Potential explanation for the decreasing birthweight trends in the second period

We found that a large proportion of the decreasing birthweight trend was explained by gestational age at delivery (i.e. length of pregnancy) similarly to other authors [18–20, 22, 25, 28]. The decreasing length of gestation over time is strongly related to the fact that the proportion of induced deliveries and caesarean sections more than doubled over the examination period. Other authors that found similar decreasing birthweight trends explained this observation by the increasing rates of early term caesarean deliveries and induced labours [6, 20, 22, 24, 25]. This is supported by the fact that births became much less likely to occur beyond gestational week 40 and much more likely to occur during weeks 37–39 [7]. In addition to shorter pregnancies, some authors proposed that decreased foetal growth per se explain part of the decreasing birthweight trend [22, 23].

It is plausible that the worsening short term pregnancy outcomes associated with advanced maternal age is compensated by early term pregnancies [25].

However, the question remains how the approach to early term deliveries will modify long-term consequences. It is known that caesarean sections are associated with an increased risk of severe acute maternal morbidity and mortality, and a higher risk of adverse outcomes in subsequent pregnancies [57]. In terms of newborn outcomes, caesarean sections are associated with increased risks of foetal respiratory problems [58] and long-term consequences (i.e. asthma, overweight, obesity, allergy) [57].

#### Subgroups driving increasing and decreasing birthweight trends

We found the fastest increase in birthweight among the oldest mothers (≥ 35 years of age), among those with multiparity, and among newborns delivered by a caesarean section in the first part of the observation period. These findings may suggest that the approach to deliveries was reactive by obstetricians: wait in the high-risk groups (older mothers, multiparas) for delivery induction or caesarean delivery until the foetus becomes large. This notion is supported by the Spanish observation that term newborns from caesarean deliveries were larger than from vaginal deliveries and newborns of multiparas were larges than those of primiparas [59].

We found the fastest decline in birthweight among the youngest mothers (< 25 years of age) in the second part of the observation period. Furthermore, newborns of multiparas and those of caesarean deliveries were no longer associated with faster increases in birthweights. These findings are compatible with the hypothesis of a proactive management of delivery, where pregnancy is terminated in high-risk women before foetal weight reaches abnormal levels.

### Strengths and limitations

Our analysis includes most Hungarian pregnancies with an ascertainment rate of 94.8%. The huge number of records allowed adjustment for several risk factors and to provide narrow CIs. The data entry software comes with detailed instructions that assures high quality of the collected variables [34].

Our analyses are limited in several ways. First, there is no way to measure changes in the obstetric decision-making process in official administrative data. As with other administrative databases, other limitations have to be acknowledged: no data is available regarding race, social status, bodyweight, and smoking habits – important determinants of birthweight. Although there is a possibility of misclassification, it should be noted that the Tauffer database is not used for reimbursement limiting the role of selective over- or under-reporting. The role of unmeasured confounding cannot be downplayed. It is possible that the increases and decreases in birthweights were responding to unobserved factors. Individual measures of maternal behaviours, characteristics, and other risk factors for obstetric interventions were also quite limited. Potentially key details about maternal health risk factors related to obstetric decisions (such as obesity) may also be missing. This limitation is especially relevant for our secondary objective (drivers of increasing and decreasing birthweight trends over time), and thus our results on this objective should be considered as hypothesis generating only.

## Conclusions

Given the strong association between large birthweight and an adverse metabolic profile in children and young adults [3, 4], our findings of an increasing birthweight trend between 1999 and 2008 may forecast an increased risk of cardiometabolic diseases in offsprings born in this period. Our results also suggest that the changes in birthweights in this period are mostly related to the aging of the mothers.

In contrast, after 2008, birthweights were decreasing. While these changes may reflect some beneficial effects in term of reduced perinatal morbidity [5], the long term effect of this decreasing birthweight trajectory cannot be predicted, as the trend is explained by the shorter pregnancies (lower gestational age at delivery) and not changes in other drivers of macrosomia (such as maternal age or BMI). Furthermore, the increasing trend in the age of the mothers is continuing unabated.

## Data Availability

The datasets used and/or analysed during the current study are available from the corresponding author on reasonable request.

## Abbreviations

ANOVA: Analysis of variance
BMI: Body mass index
CI: Confidence Interval
g: Gram
IQR: Interquartile range
SE: Standard error
SPSS: Statistical Package for the Social Sciences
UK: United Kingdom
US: United States
